# Exploiting upper-limb functional principal components for human-like motion generation of anthropomorphic robots

**DOI:** 10.1186/s12984-020-00680-8

**Published:** 2020-05-13

**Authors:** Giuseppe Averta, Cosimo Della Santina, Gaetano Valenza, Antonio Bicchi, Matteo Bianchi

**Affiliations:** 1 Research Center “Enrico Piaggio”, University of Pisa, Largo Lucio Lazzarino 1, Pisa, 56126 Italy; 2 Soft Robotics for Human Cooperation and Rehabilitation, Fondazione Istituto Italiano di Tecnologia, via Morego, 30, Genova, 16163 Italy; 3 Dipartimento di Ingegneria dell’Informazione, University of Pisa, Via G. Caruso, 16, Pisa, 56122 Italy; 4 Computer Science and Artificial Intelligence Laboratory, Massachusetts Institute of Technology, 32 Vassar st, Cambridge, 02139 MA USA

**Keywords:** Functional principal components, Human-robot interaction, Rehabilitation robotics, Assistive robotics, Companion robots, Exoskeletons

## Abstract

**Background:**

Human-likeliness of robot movements is a key component to enable a safe and effective human-robot interaction, since it contributes to increase acceptance and motion predictability of robots that have to closely interact with people, e.g. for assistance and rehabilitation purposes. Several parameters have been used to quantify how much a robot behaves like a human, which encompass aspects related to both the robot appearance and motion. The latter point is fundamental to allow the operator to interpret robotic actions, and plan a meaningful reactions. While different approaches have been presented in literature, which aim at devising bio-aware control guidelines, a direct implementation of human actions for robot planning is not straightforward, still representing an open issue in robotics.

**Methods:**

We propose to embed a synergistic representation of human movements for robot motion generation. To do this, we recorded human upper-limb motions during daily living activities. We used functional Principal Component Analysis (fPCA) to extract principal motion patterns. We then formulated the planning problem by optimizing the weights of a reduced set of these components. For free-motions, our planning method results into a closed form solution which uses only one principal component. In case of obstacles, a numerical routine is proposed, incrementally enrolling principal components until the problem is solved with a suitable precision.

**Results:**

Results of fPCA show that more than 80% of the observed variance can be explained by only three functional components. The application of our method to different meaningful movements, with and without obstacles, show that our approach is able to generate complex motions with a very reduced number of functional components. We show that the first synergy alone accounts for the 96% of cost reduction and that three components are able to achieve a satisfactory motion reconstruction in all the considered cases.

**Conclusions:**

In this work we moved from the analysis of human movements via fPCA characterization to the design of a novel human-like motion generation algorithm able to generate, efficiently and with a reduced set of basis elements, several complex movements in free space, both in free motion and in case of obstacle avoidance tasks.

## Introduction

There are many examples in literature that have highlighted the importance of human-likeness (HL) to ensure a safe and effective Human-Robot Interaction (HRI) [1, 2]. This aspect has gained increasing attention, since it could open interesting perspectives for the control of artificial systems that closely interact with humans, as is the case of assistive, companion and rehabilitative robots. For the latter category, for example, human-inspired movement profiles - which are characterized by i) low jerk values at the Cartesian or joint level and ii) bell-shaped velocity profiles (see [3–5]) - could be used as reference trajectories for rehabilitation exoskeletons ([6–9], see also [10, 11] for review), as an alternative to, and/or in association with, classic rehabilitation procedures [12–14].

Indeed, the motion of a robot that shares its environment with humans can be more easily *predicted*, and hence accepted, by the user, if its movements are designed taking inspiration from actual human movements [1, 15], leading to a general enhancement in terms of system usability and effectiveness, especially in assistive robotics applications [16–18]. However, the design of control laws, which effectively ensure human-like behavior in robotic systems, is not straightforward, representing an important topic within the general framework of robot motion planning.

Usually, HL is achieved leveraging on a vast neuroscientific literature to devise cost functions (see [3]), whose optimization introduces HL characteristics in the motion. For example, in [26] human-like artificial motions were generated through jerk minimization, while in [33] the Authors exploited the minimization of joint torques, and in [27] the Virtual Spring-Damper Hypothesis was proposed. Neural Networks have also been used for human-like character animation [29–31]. However, optimization-based methods usually come with hypotheses on motion generation that may limit the variability of the planned movement and, sometimes also lack experimental support [34]. On the other hand, learning methods typically require a large dataset whose dimensionality dramatically increases with task complexity.

To the best of authors’ knowledge, a direct exploitation of human observations for robotic arm motion generation has not been applied yet. This approach would come with several advantages, since human-likeness would be intrinsically guaranteed. However, a mere copy-cutting from nature would be unfeasible, and clearly a daunting task. What we propose instead is to use neuromechanistic data, intended here in terms of time-modulation of joint angular values, and model them with a mathematical language, which can be easily understood and effectively implemented in an artificial body. A notable example of this approach is represented by the concept of hand postural synergies, which was mathematically modelled in [35], and then successfully exploited for the design and control of robotic end-effectors and for grasp planning [36–38].

In this work, we propose to directly embed human upper limb principal motion modes for the planning of anthropomorphic manipulators. To this end, we recorded and organized the joint trajectories of the arm of human subjects performing a set of Daily Living Activities (ADLs) to build a comprehensive dataset. We then applied statistical analysis [39] (namely functional Principal Component Analysis, fPCA) to extract a reduced number of basis functions, or *functional Principal Components*, which explain, for each joint, most of the trajectory variability. As reported later, our results show that a weighted sum of only three functional components takes into account more than 80% of the total variance at joint level.

Capitalizing on these results, we then formulate the planning problem - for a given anthropomorphic manipulator - as an optimization problem. More specifically, the final motion of the manipulator is obtained by solving an optimization problem in a latent space defined by the weights of the functional Principal Components. The core idea of the proposed approach is to use the functional Principal Components extracted from the observations of human movements as basis elements, whose combination is used to optimize the generation of any point-to-point trajectory of the arm in a dummy human. For free-motions, our method results into a closed form solution, which uses only one functional Principal Component.

This methodology comes with a significant perspective shift: from the search for optimal paths to the identification of a reduced number of scalars weighting the functional components. This could enable to rapidly achieve a solution for the planner, which is intrinsically human-like. To further increase the cost-effectiveness of our method, we propose an incremental enrollment of the functional components, as suggested in [40]. In this manner, the number of functional Principal Components needed to perform the task is tailored on task complexity, avoiding the useless inclusion of higher order functional Principal Components.

We demonstrate in simulations that our techniques can generate human-like motion using a reduced number of functional components. The human-likeliness of the generated movements is evaluated according to the indexes reported in [3, Appendix] observing the velocity profiles and jerk values [4, 41].

To conclude, this paper contributes with: i) an extensive study on human upper limb functional Principal Components, which we have pursued by applying our functional analysis approach reported in [39] to a dataset obtained by enrolling a substantially increased number of participants (33) in the experiments; ii) a new methodology for human-like motion generation, which we have applied to the case of point-to-point motions with and without obstacle avoidance, which intrinsically embeds the principal modes of human upper limb motions; iii) simulation results, that show the effectiveness of our method.

## Methods

### Functional principal components of upper limb

In a previous work, we presented a functional characterization of upper-limb movements during Activities of Daily Living (ADLs) organized in a dataset that comprised seven subjects. We showed that a reduced-dimensionality set of functional Principal Components (fPCs) accounts for a large part of upper-limb movement variability at joint level [39].

**Fig. 1:**
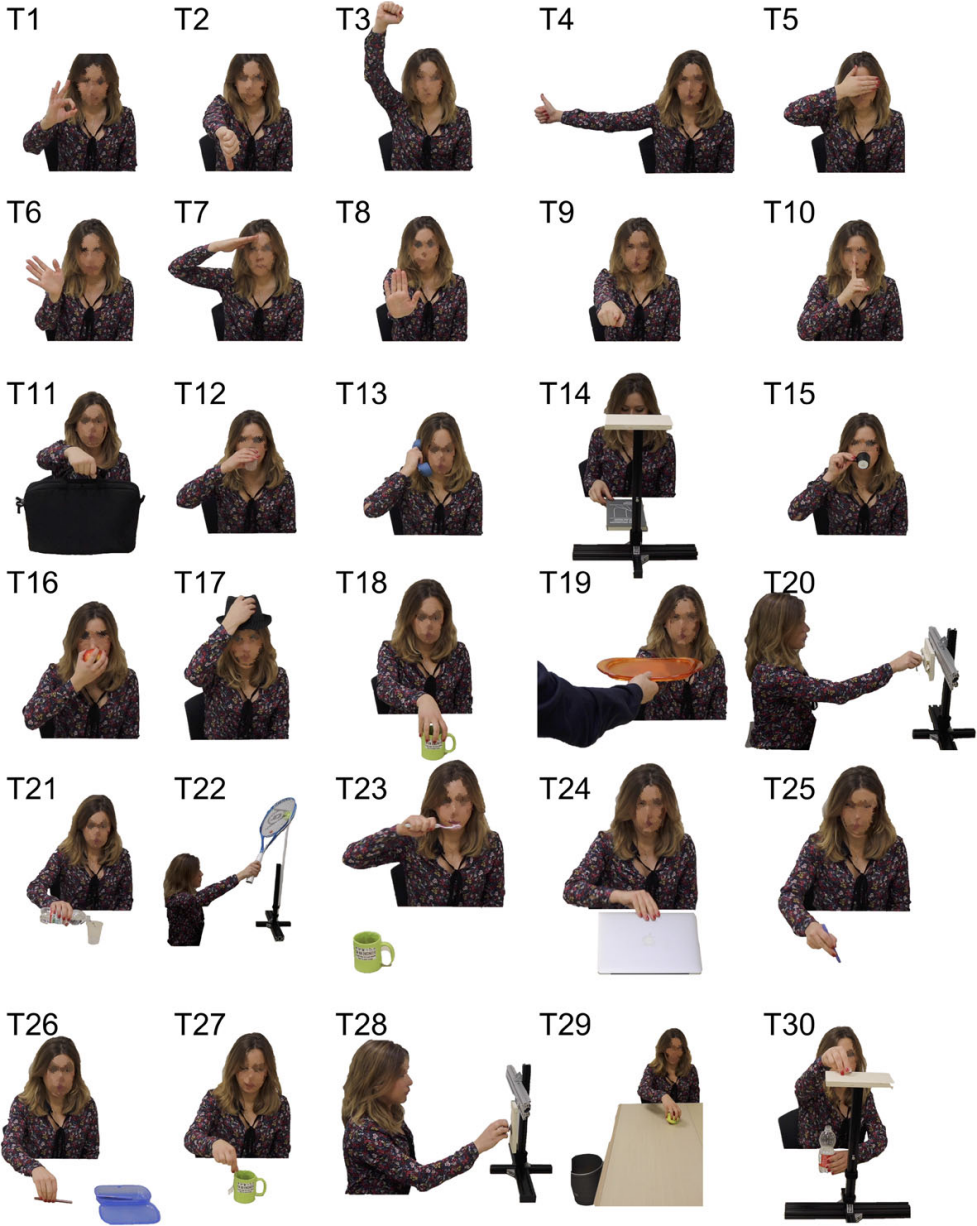
From top to bottom, and from left to right, pictures of the tasks considered in this study. Verbal description of the tasks is reported in Table 5

To expand and confirm the validity of our analysis, in this work we built a completely new and extended dataset of ADLs, which includes movements recorded from 33 healthy subjects (17 women, 26.6 y.o. on average). The experimental protocol consisted of 30 activities of daily living as in [39]. Each activity was repeated three times for a grand total of 90 tasks per person. All experimental procedures were approved by the local Ethical Committee of the University of Pisa. The movements were grouped into three categories, characterized by a different kind of interaction with external objects: *Intransitive*, i.e. gestures; *Transitive*, i.e. movements that involve the interaction with one object; *Tool-Mediated*, i.e. movements in which one object is used to interact with another one. Please refer to the Appendix of this paper for a textual description of the tasks, and to Fig. 1 for their graphical representation. Note that each subject was instructed about the task to accomplish but not on the specific motion to perform for achieving the task goal.

#### Motion identification

**Fig. 2:**
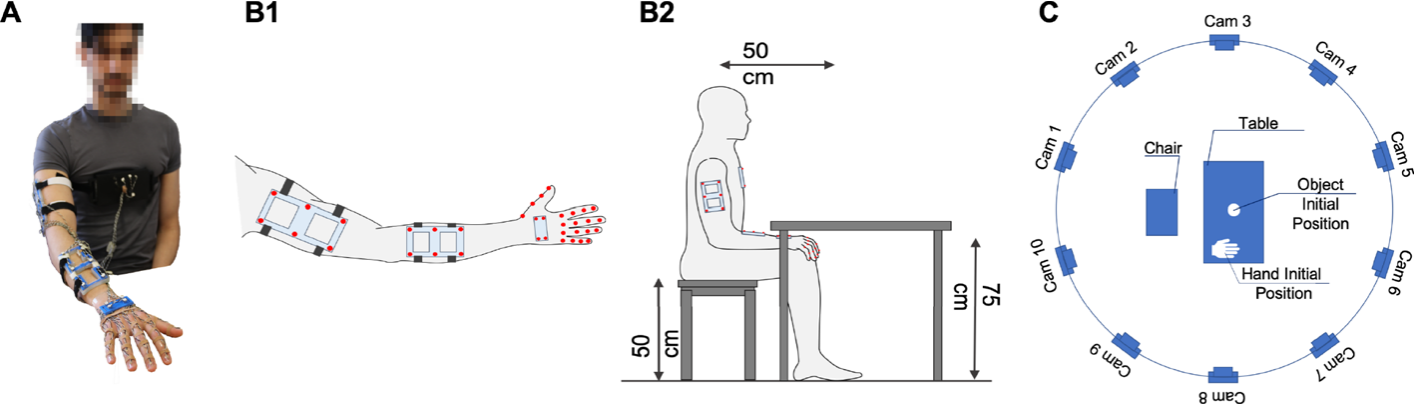
Schematics of the experimental setup. In Subfigure A we show a picture of a subject wearing markers on the upper limb. Subfigures B and C detail the experimental setup. Arm and forearm are tracked using six markers each, while the hand is tracked using four markers. Four additional markers are placed on the subject’s chest to track the reference. Markers are redundant and fastened on rigid supports. 10 stereo-cameras are placed around the scene to minimize marker occlusion

Movements were recorded using a 3D motion tracking system with active markers (Phase Space). Ten stereo-cameras working at 100Hz tracked the 3D position of active markers, which were placed on rigid supports and attached to upper limb links (see Fig. 2).

**Fig. 3:**
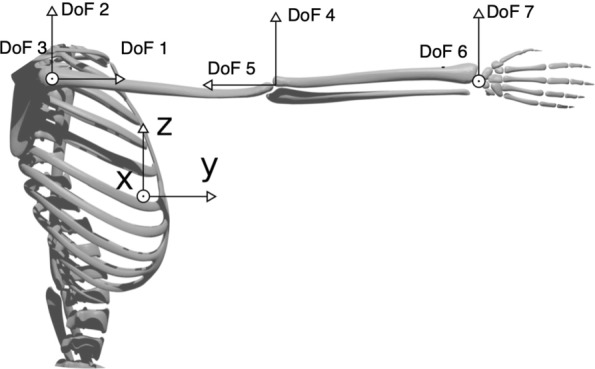
Kinematic parametrization, the labels DoF 1, $\dots $, DoF 7 refer to the joint angles of the model

To map the recorded movements on an analytic kinematic model, we developed a two-phase procedure, which consisted of a preliminary calibration of the model, followed by a Kalman-based identification. In this work we used the same kinematic model discussed in [39], i.e. a serial manipulator with three rigid links connected by seven revolute joints (see Fig. 3). This model can be completely defined using 14 parameters: 2 parameters for bone length and 12 parameters to identify the position of the markers with respect to the kinematic chain (more technical details can be found in [39]). This set of parameters (*p*_*G*_) was calibrated, for each subject, by solving the following optimization problem:

1 $$ (q^{*}, p^{*}_{G}) = \text{arg}\min_{q_{k} \in D_{q}, p_{G} \in D_{p}} \frac{1}{2} \sum_{k=1}^{N_{p}} r^{T}_{k} r_{k},  $$

where *r*_*k*_ is the residual function:

2 $$ r_{k}(q_{k},p_{G}) = y_{k} - f(q_{k},p_{G})  $$

at time frame *k*, calculated as the difference between the measured position of markers *y*_*k*_ and their estimation via Forward Kinematics (FK) *f*(*q*_*k*_,*p*_*G*_). The FK is function of *q*_*k*_, i.e. the current estimation of joint angles, and *p*_*G*_, i.e. the parameters of the kinematic model. The optimization problem is constrained by *D*_*q*_, i.e. the range of motion of the joints, and *D*_*p*_, i.e. the maximum variation of parameters with respect to a preliminary manual estimation. *N*_*p*_ refers to the number of samples used for the calibration – 10 equally time-spaced samples in our case, as in [39, 42]. Given the calibrated vector *p*_*G*_ for the specific subject, the upper limb pose is completely described by 7 joints angles $[q_{1}, \dots, q_{7}]^{T}$.

The calibrated model was then used as a component for a Kalman-based identification procedure. More specifically, if we consider, at time frame *k*, the joints angle vector *q*_*k*_ as the state of a stochastic process with *w*_*k*_ and *v*_*k*_ process and observation zero mean Gaussian noises, with covariance *Q*_*k*_ and *R*_*k*_ respectively, we have that the system can be written as

3 $$ \left\{\begin{array}{l} q_{k} = q_{k-1} + w_{k}\\ y_{k} = f(q_{k}) + v_{k} \end{array}\right.  $$

Recursively, the state *q*_*k*_ can be estimated from *q*_*k*−1_ with the following implementation of a Kalman filter [43]: *Prediction* of the future state $\hat {q}_{k|k-1} = \hat {q}_{k-1}$; *Update* of the state estimation as $\hat {q}_{k|k} = \hat {q}_{k|k-1} + K_{k}\tilde {r}_{k}$. The prediction correction is the product between the residual values vector $\tilde {r}_{k} = y_{k} -f(\hat {q}_{k|k-1})$ and the Kalman Gain *K*_*k*_, defined as product between the covariance matrix estimation of the predicted state *P*_*k*|*k*−1_, the Jacobian matrix $H_{k} = \frac {\partial (f(q))}{\partial q}$ and the inverse of the residual covariance.

Finally, to effectively and jointly process different acquisitions with different temporal lengths, a time normalization is required. To this end, we used Dynamic Time Warping. More specifically, given a reference signal extracted from the dataset *q*_*ref*_(*t*), all the trajectories were normalized in time with respect to (w.r.t.) the reference one, by solving the following minimization problem

4 $$ \begin{aligned} (S_{i},T_{i}) = \text{arg}\min_{S_{i}>0,T_{i}} (|| q_{ref}(t) - q_{i}(S_{i}t - T_{i}) ||), \end{aligned}  $$

where *S*_*i*_ and *T*_*i*_ are the time-stretch and the translation parameter respectively.

#### Functional analysis

In the following, we will briefly summarize the main theoretical concepts behind functional Principal Component Analysis (fPCA), and discuss their application to the investigation of human upper limb motions. Let us consider a generic upper-limb movement $q(t) : \mathbb {R} \rightarrow \mathbb {R}^{7},$ where *t*∈[0,*t*_fin_] is the time variable, and 7 is the number of the upper limb degrees of freedom (DoFs) of the kinematic model we considered for our analyses. The goal of fPCA targets the identification of a suitable reduced functional representation, which can closely approximate *q*(*t*) (joint trajectories). For these reasons, fPCA can be regarded as a functional extension in the temporal domain of Principal Component Analysis (PCA). Indeed, as Principal Components identify inter-joint couplings that account for most kinematic pose variability, functional PCs (fPCs) are functions that allow to describe most of the movement variability over time, at joint level. Using the fPCA framework, a generic upper limb motion *q*(*t*) can be described as a weighted sum of a set of base functions *S*_*i*_(*t*), or functional Principal Components, that is:

5 $$ \begin{aligned} q(t) \simeq \bar{q} + S_{0}(t) + \sum_{i = 1}^{s_{\text{max}}} \alpha_{i} \circ S_{i} (t) \;, \end{aligned}  $$

where $ \alpha _{i} \in \mathbb {R}^{\mathrm {n}}$ is a vector of weights. *n* is the dimension of *q*(*t*), in our case equal to 7. $S_{i} (t)\in \mathbb {R}^{\mathrm {n}}$ is the i^th^ basis element and *s*_max_ is the number of basis elements. The operator ∘ is the Hadamard product, i.e. the element-wise product [44]. $\bar {q} \in \mathbb {R}^{7}$ is the average posture of *q* evaluated as

6 $$ \bar{q} = \int_{0}^{t_{\text{fin}}} q(\tau) \mathrm{d}\tau \;,  $$

while $S_{0} : \mathbb {R} \rightarrow \mathbb {R}^{7}$ is the *zero-order* synergy, i.e. the average trajectory across all the trajectories *q* in the dataset, for all the tasks and subjects.

fPCA is used to identify a basis of functions $\{S_{1},\dots,S_{s_{\text {max}}}\}$ that maximizes the explained variance of the movements in the collected dataset. The first fPC *S*_1_(*t*) is the function that solves the following problem

7 $$ \begin{aligned} &\!\min_{S_{1}} &\quad& \sum_{j=1}^{R} \left(\int S_{1}(t) q_{j}(t) dt \right)^{2}\\ &\text{subject to} & &||S_{1}(t)||^{2}_{2} = \int_{0}^{t_{\text{fin}}} S_{1}^{2}(t) dt = 1 \;, \end{aligned}  $$

where *R* is the number of trajectories that compose our dataset. The other fPCs *S*_*i*_(*t*) are the functions that solve the following constrained optimization problems

8 $$ {}\begin{aligned} &\!\min_{S_{i}} &\quad& \sum_{j=1}^{R} \left(\int S_{1}(t) q_{j}(t) dt \right)^{2}\\ &\text{subject to} & &||S_{i}(t)||^{2}_{2} = 1 \\ & & & \int_{0}^{t_{\text{fin}}} S_{i}(t) S_{k}(t) dt = 0 \,, \; \forall k \in \{1, \dots, i-1\}\;. \end{aligned}  $$

This results in an ordered set of functions that - given a number of basis elements *s*_max_ - maximizes the explained variability of the joint trajectories of the dataset. This opens the possibility to use these functions as a *smart basis* to generate HL movements in a latent space. In the remaining of this work, we present an algorithm that exploits functional Principal Components extracted from the analysis of human upper limb movements for the generation of point-to-point robotic/artificial motions, with and without the presence of obstacles along the trajectory. These movements naturally embed HL characteristics, (see next section for the proposed method).

To test the effectiveness of this approach, we performed simulations of both free-motion and obstacle-avoidance cases. We used a robotic arm simulator with the same kinematic description we used during the experiments with human subjects. Hereinafter we refer to this simulator as *dummy human*. We considered four motions that are representative of the upper limb movement workspace (see the Results section): T.1) accounts for right-to-left side motions of the trunk, which typically sub-serve the execution of actions for maneuvering objects on a table; T.2) is representative of movements towards a position in front of the subject. T.3) relates to arm elevation towards a position upon the subject’s head; T.4) deals with reaching movements toward the subject’s own face, which are usually executed for self-care tasks, e.g. self-feeding etc. Numerical values of the 7 joint angles in the initial and desired final conditions, *q*_0_ and *q*_fin_, respectively, for each of the four tasks are reported in Table 1.

**Table 1:** Initial (*q*_0_) and final (*q*_fin_) configurations for the four tasks considered

Task		dof 1	dof 2	dof 3	dof 4	dof 5	dof 6	dof 7
T.1	*q*_0_	−0.31	−0.58	−0.8	0.47	−1.23	0.22	0.65
	*q*_fin_	−0.33	0.34	−0.39	0.44	−0.69	0.36	0.15
T.2	*q*_0_	0.09	−0.79	−0.62	0.65	−1.50	0.06	0.76
	*q*_fin_	0.65	−0.40	0.07	0.79	−1.32	0.87	0.63
T.3	*q*_0_	−0.48	−0.07	−0.71	0.03	−0.86	0.52	0.47
	*q*_fin_	−1.67	−0.68	−0.33	−0.21	−0.09	−0.26	0.52
T.4	*q*_0_	−0.28	−0.86	−0.92	0.86	−1.52	−0.12	0.77
	*q*_fin_	0.65	−0.78	−0.12	2.18	−1.52	0.57	0.59

Angles are expressed in radians

In our simulations, we considered three scenarios for each task: no obstacle (i.e. free motion), one obstacle, and two obstacles. In the latter two scenarios, we decided to place the obstacles along the optimal trajectories computed for the free-motion case. In this manner, we forced the algorithm to modify the initial guess. Details on the obstacle location – with respect to the Inertial System of Reference placed as in Fig. 3 – and dimension (expressed in terms of the center and radius of a sphere surrounding the obstacle, respectively) are reported in Table 2.

**Table 2:** Details on the obstacle location *Position* – with respect to the Inertial System of Reference placed as in Fig. 3 – and dimension *Radius* (expressed in terms of the center and the radius of a sphere surrounding the obstacle, respectively) considered during the simulations, for the one obstacle (*1 Obs*) and two obstacle (*2 Obs*) case, for the different tasks (*T.1, T.2, T.3, T.4*)

	1 Obs	2 Obs		
	Position	Radius	Position	Radius
T.1	[-167 200 323]	90	[-167 200 323]	40
			[ -89 96 436]	40
T.2	[0 220 350]	50	[0 220 350]	50
			[70 170 135]	75
T.3	[313 480 321]	70	[313 480 321]	70
			[-2 311 353]	70
T.4	[-35 202 310]	40	[-35 202 310]	40
			[-168 158 322]	40

These quantities are expressed in [mm]

## Motion generation via functional principal components

As discussed in the Introduction, typical approaches used in literature to achieve human likeness [26] in robotic motions rely on the strong assumption that human movements are generated by optimizing a known cost function $J_{\text {hl}}(q):\mathrm {C}_{7}^{1}[0,t'_{\text {fin}}) \rightarrow \mathbb {R}^{+}$, where $\mathrm {C}_{7}^{1}[0,t'_{\text {fin}})$ is the space of smooth functions going from [0,*t*fin′) to the joint space $\mathbb {R}^{7}$, and $t^{\prime }_{\text {fin}}$ is the final time, which in general will be different from *t*_fin_ as defined in the previous section. The function *J*_hl_ is used to produce artificial natural motions by solving the problem

9 $$  \min_{q \in \mathrm{C}_{7}^{1}[0,t'_{\text{fin}})} \quad J_{\text{hl}}(q) \;.  $$

How to choose *J*_hl_ is not obvious, and it is indeed a very debated topic in literature. However only achieving human likeness is meaningless without specifying also a task to be accomplished.

For this reason also a model of the task should be added to (9). We formulate the latter point in terms of the minimization of an additional cost function $J_{\text {task}}:\mathrm {C}_{7}^{1} \rightarrow \mathbb {R}^{+}$. As soon as the need for minimizing *J*_task_ is introduced, (9) becomes a multi-objective optimization, which is of very difficult formulation and solution, except for very simple cases [26].

In this work, we propose an approach that allows to by-pass this issue. Instead of using data to guess a reasonable *J*_hl_(·), and then explicitly optimize it, we propose to directly embed human likeness in the choice of the functional subspace where the optimization occurs. More specifically, we move from the infinite dimensional functional space $\mathrm {C}_{7}^{1}[0,t'_{\text {fin}})$, to its finite dimensional subspace containing all the functions so constructed:

10 $$ q(t) = \bar{q} + S_{0}(t'(t)) + \sum_{i = 1}^{M} \alpha_{i} \circ S_{i} (t'(t))  $$

with $\bar {q},S_{i},\alpha _{i}$ defined as in the previous section and *t*^′^ is a linear warping w.r.t. the definition of time used in the previous section, i.e. $t'(t) = \frac {t'_{\text {fin}}}{t_{\text {fin}}} t$. In this way the principal components can be used to generate motions happening within any time horizon [0,*t*fin′).

*M*≤*s*_max_ is the number of functional Principal Components considered in the optimization (with *s*_max_ as in (5)). According to the results preliminary presented in [39] and further extended and confirmed in this paper, it is plausible to expect that a low number of functional Principal Components should be sufficient to implement most of the human-like motions at the joint level. Therefore the multi-object and unconstrained optimization can be formulated as the following constrained optimization problem:

11 $$ \begin{aligned} &\!\min_{\bar{q}, \alpha_{1},\dots,\alpha_{M}} &\qquad& J_{\text{task}}(q)\\ &\text{subject to} & &q(t') = \bar{q} + S_{0}(t') + \sum_{i = 1}^{M} \alpha_{i} \circ S_{i} (t') \;. \end{aligned}  $$

In this manner, we can narrow the search space, with the twofold purpose of ensuring human likeness, and strongly simplifying the control problem (indeed, the search space is now of dimension *M*+1). In the following subsections we present the application of this approach, tailoring *J*_task_ on the generation of simple point-to-point free movements, as well as more complex motions with obstacle avoidance.

### Point-to-Point free motions

We propose here to generate a human-like point-to-point motion by solving the following optimization problem, instance of the more general formulation (11)

12 $$ \begin{aligned} &\!\min_{\bar{q}, \alpha_{1},\dots,\alpha_{M}} &\qquad& || q(0) - q_{0} ||^{2}_{2} + || q(t'_{\text{fin}}) - q_{\text{fin}}||^{2}_{2}\\ &\text{subject to} & &q(t') = \bar{q} + S_{0}(t') + \sum_{i = 1}^{M} \alpha_{i} \circ S_{i} (t') \;, \end{aligned}  $$

where *q*(0) and *q*(*t*fin′) are the initial and final poses of the calculated trajectory, while *q*_0_ and *q*_fin_ are the desired initial and final poses respectively. In this simple case, a single functional Principal Component (i.e. *M*=1) is already sufficient to solve (12) with zero error.

We start by imposing the zero error conditions *q*_0_=*q*(0),*q*_fin_=*q*(*t*fin′), which can be rewritten by using (10)

13 $$  \begin{aligned} q_{0} &= \bar{q} + S_{0}(0) + \alpha_{1} \circ S_{1} (0) \\ q_{\text{fin}} &= \bar{q} + S_{0}(t'_{\text{fin}}) + \alpha_{1} \circ S_{1}(t'_{\text{fin}}) \;. \end{aligned}  $$

We subtract the second equation from the first, and exploit the associativeness of Hadamard product, to obtain

14 $$ \begin{aligned} &q_{\text{fin}} - q_{0} = S_{0}(t'_{\text{fin}}) - S_{0}(0) + \alpha_{1} \circ (S_{1}(t'_{\text{fin}}) - S_{1}(0)), \end{aligned}  $$

which implies

15 $$ \begin{aligned} {} \alpha_{1} &= [(q_{\text{fin}} \!- q_{0}) - (S_{0}(t'_{\text{fin}}) - S_{0}(0))] \circ (S_{1}(t'_{\text{fin}}) - S_{1}(0))^{\circ -1} \end{aligned}  $$

where ^∘−1^ is the Hadamard inverse, as defined in [44]. Substituting (15) back into (13) yields

16 $$ \begin{aligned} \bar{q} &= q_{0} - \alpha_{1} \circ S_{1} (0) \\ &= q_{0} - [[(q_{\text{fin}} - q_{0}) - (S_{0}(t'_{\text{fin}}) - S_{0}(0))] \\ & \qquad \circ (S_{1}(t'_{\text{fin}}) - S_{1}(0))^{\circ -1}] \circ S_{1} (0) \;, \end{aligned}  $$

which fully specifies the trajectory that performs the point to point motion while being human-like.

#### Obstacle avoidance

Let us consider the case in which we also need to avoid one or more obstacles, while performing the point-to-point motion. We can generalize the generation of the human-like trajectory as

17 $$ \begin{aligned} &\!\min_{\bar{q}, \alpha_{1},\dots,\alpha_{M}} &\qquad& \left|\left| \left[\begin{array}{l} q(0) - q_{0} \\ q(t'_{\text{fin}}) - q_{\text{fin}} \end{array}\right] \right|\right|_{2}^{2} + \rho P(q, P_{O})\\ &\text{subject to} & &q(t') = \bar{q} + S_{0}(t') + \sum_{i = 1}^{M} \alpha_{i} \circ S_{i} (t') \;. \end{aligned}  $$

Two terms can be distinguished in this cost function. The first contribution guarantees that the desired initial and final poses are achieved, as for the free motion case (12). The second term takes into account the distance w.r.t. obstacles. For the sake of conciseness, and without any loss of generality, we assume here *N*_O_ spherical obstacles. We call $P_{\mathrm {O}} = \{P_{\mathrm {O}_{1}},\dots,P_{\mathrm {O}_{N_{\mathrm {O}}}}\}$ the set containing the Cartesian coordinates of all the centers of these obstacles.

*P*(*q*,*P*_*O*_) is a potential-based function [45] that sums up, for each obstacle, a term inversely proportional to the minimum distance between the obstacle and the closest joint trajectory, i.e.

18 $$ P(q, P_{O}) = \sum_{i = 1}^{N_{O}} \frac{1}{m_{i}(q([0,t'_{\text{fin}}}]), P_{\mathrm{O}_{i}})^{2}  $$

where *m*_*i*_ is the distance between the arm and the *i*−*th* obstacle, defined as

$$m_{i}(q([0,t'_{\text{fin}}]), P_{\mathrm{O}_{i}}) = \min_{k} \{ d(h_{k}(q([0,t'_{\text{fin}}])), P_{\mathrm{O}_{i}})\} \;.$$

The distance between the *k*−*th* point of contact with forward kinematics *h*_*k*_, and the *i*−*th* sphere is

19 $$ \begin{aligned} d(h_{k}(q([0,t'_{\text{fin}}])), P_{\mathrm{O}_{i}}) = \max\left\{\min_{x \in h_{k}(q([0,t'_{\text{fin}}]))} ||P_{\mathrm{O}_{i}} - x||_{2}, R_{\mathrm{O}_{i}}\right\} \;, \end{aligned}  $$

with $R_{O_{i}}$ radius of the sphere.

#### Incremental optimization procedure

The problem of motion generation with obstacle avoidance does not have a closed-form solution, hence the optimal trajectory is calculated via numerical optimization. To do this, we took inspiration by the ordering of fPCs basis, according to a descending amount of the associated explained variance, and implemented an incremental procedure reported in Algorithm blue1.


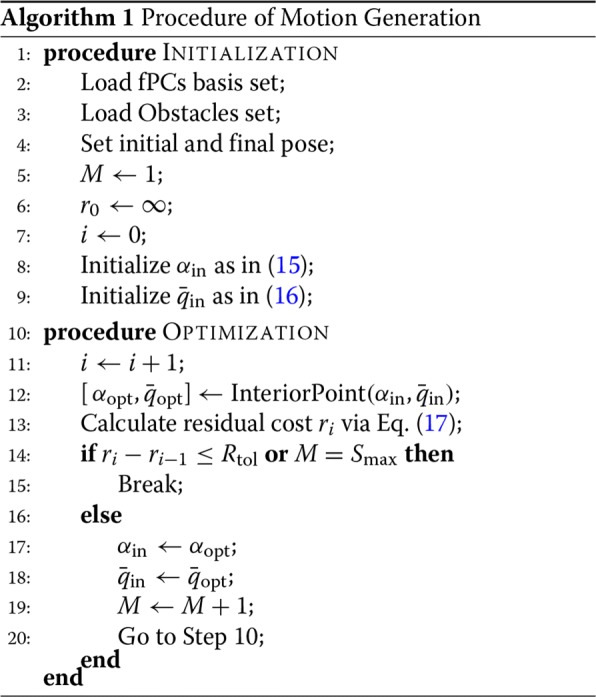


The proposed approach calculates for each step the optimal trajectory that minimizes the error in starting and final position while maximizing the distance from the obstacles. We consider as initial condition the one specified by (15) and (16). If the corresponding solution is sufficiently far from the obstacles, this choice already defines the globally optimal solution. If the obstacles are not very close to the aforementioned trajectory, then solving (17) with *M*=1 would fine tune the initial guess, achieving good results.

In case of obstacles very close to or even intercepting the free-motion trajectory, at least one more fPC should be enrolled to suitably solve the problem. The more are the basis elements enrolled, the more complex are the final trajectories that can be implemented. However, a higher cardinality of enrolled elements usually comes with a larger computational cost and dramatically increases the number of local minima, thus boosting the complexity of the problem.

## Results

### Functional principal components of upper limb motion

**Fig. 4:**
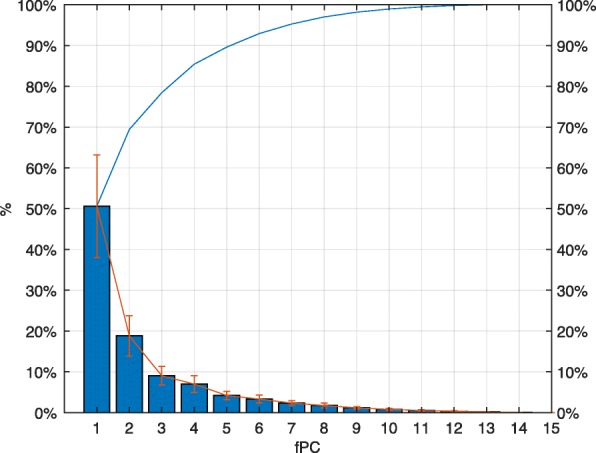
Average variance explained by each fPC (blue bars) ± standard deviation (in red). The blue line on top reports the incremental sum of mean values

**Fig. 5:**
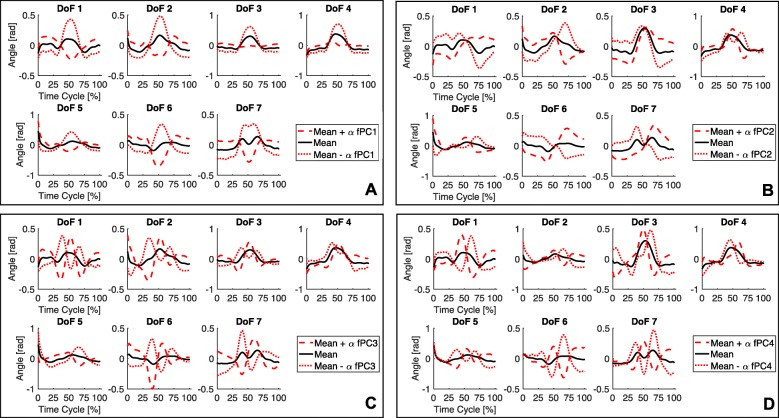
From figure A to D, mean trajectory (in black) and the contribution (signed) of each functional Principal Component, from fPC1 to fPC4 respectively. Components ordering follows the indexing of Fig. 3

In Fig. 4, we report, for each joint, the functional Principal Components over the time. As shown in Fig. 5, the first four fPCs together (i.e. a weighted sum of them) account for around the 85% of the total variance of the dataset.

What is also noticeable is that lower order functional Principal Components (i.e. fPC1 and fPC2) introduce low-frequency contributions to the mean function, while higher order functional Principal Components add higher-frequency terms to the signal. In other terms, this behavior suggests that lower order functional Principal Components are mainly devoted to a gross definition of the whole trajectory, while higher order components deal with the fine tuning of the specific movement. Note that these findings are totally coherent and, hence, further confirm the outcomes of [39].

### Human-Like motion generation: validation

**Fig. 6:**
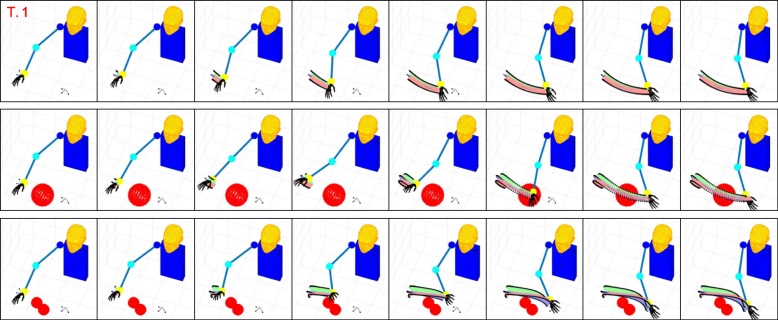
From top to bottom, the reconstruction for task 1 is reported for the free motion case, the single obstacle avoidance and the double obstacle avoidance case respectively. For each row, motion execution is intended from left to right and time-frames are evenly spaced. Reference system at wrist level is finally plotted along the whole trajectory

**Fig. 7:**
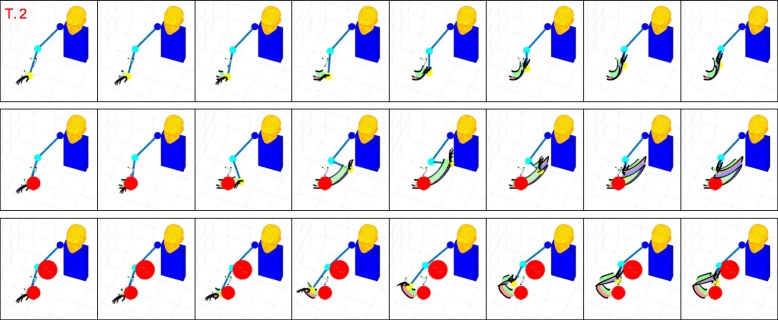
From top to bottom, the reconstruction for task 2 is reported for the free motion case, the single obstacle avoidance and the double obstacle avoidance case respectively. For each row, motion execution is intended from left to right and time-frames are evenly spaced. Reference system at wrist level is finally plotted along the whole trajectory

**Fig. 8:**
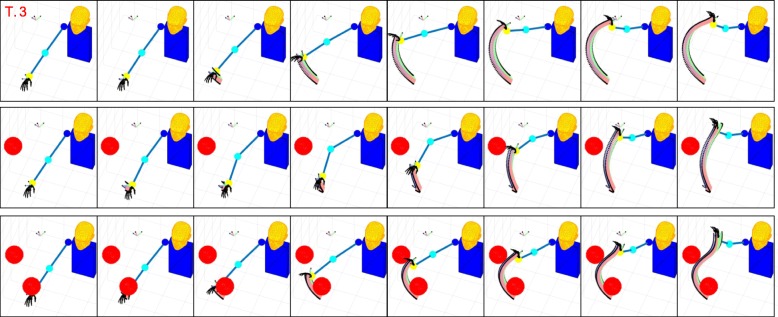
From top to bottom, the reconstruction for task 3 is reported for the free motion case, the single obstacle avoidance and the double obstacle avoidance case respectively. For each row, motion execution is intended from left to right and time-frames are evenly spaced. Reference system at wrist level is finally plotted along the whole trajectory

**Fig. 9:**
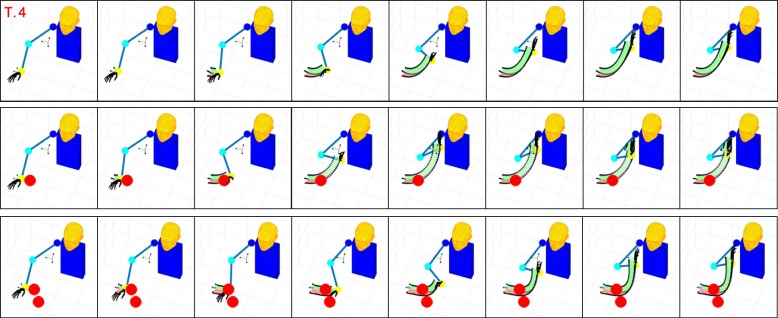
From top to bottom, the reconstruction for task 4 is reported for the free motion case, the single obstacle avoidance and the double obstacle avoidance case respectively. For each row, motion execution is intended from left to right and time-frames are evenly spaced. Reference system at wrist level is finally plotted along the whole trajectory

**Fig. 10:**
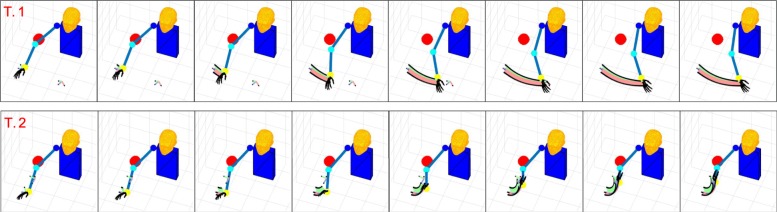
From top to bottom, the reconstruction for task 1 and 2 are reported in case of obstacles on the elbow. For each row, motion execution is intended from left to right and time-frames are evenly spaced. Reference system at wrist level is finally plotted along the whole trajectory

Figures 6, 7, 8, and 9 present graphical representations of the human-like trajectories generated by the proposed method, from T.1 to T.4. The first row of each figure depicts the free motion case, the second row the one-obstacle case, and the third the two-obstacle one. In all the considered cases the result is a smooth trajectory connecting the two postures without interacting with the obstacles. This is also evident from Fig. 10, where we present the profiles of angles and angular velocities over time.

Table 3 reports, for each task and each environment, the number of functional Principal Components retrieved by Algorithm blue1. As expected, in the zero-obstacle case, all tasks can be executed using only one fPC. When obstacles are introduced, the number *M* of functional Principal Components increases. However, the complex actions that we can observe in Figs. 6, 7, 8, and 9 can be performed with a maximum of three functional Principal Components. Numerical values of the minimum distance (expressed as Euclidean norm) between the manipulator and the obstacles in the simulations, considering both the free motion case and the Obstacle Avoidance part, are reported in Table 4.

**Table 3:** # of functional Principal Components (*M*) enrolled by Algorithm blue1

Number of				
Obstacles	Task 1	Task 2	Task 3	Task 4
0	1	1	1	1
1	2	2	2	2
2	3	2	2	2

**Table 4:** Minimum distance (in [mm]) between the manipulator and the obstacles with (*With OA*) and without (*Without OA*) the Obstacle Avoidance (OA) part, for *1 Obs* and *2 Obs* case and the different tasks *T.1, T.2, T.3, T.4*

	1 Obs		2 Obs	
	Without OA	With OA	Without OA	With OA
T.1	9.5	164	(9.5,129.5)	(164,147)
T.2	18	86.5	(18,75)	(170,92)
T.3	33.5	157	(33.5,121)	(117.5,107.5)
T.4	48	88.5	(48,119.5)	(88.5,122.5)

**Table 5:** List of Movements

#	# Cut	Class	Description
1		Int	Ok gesture (lifting hand from the table)
2		Int	Thumb down (lifting hand from the table)
3		Int	Exultation (extending the arm up in the air with closed fist)
4		Int	Hitchhiking (extending the arm along the frontal plane, laterally, parallel to the floor, with extended elbow, closed fist, extended thumb)
5		Int	Block out sun from own face (touching the face with the palm and covering the eyes)
6		Int	Greet (with open hand, moving wrist) (3 times)
7		Int	Military salute (with lifted elbow)
8		Int	Stop gesture (extending the arm along the sagittal plane, parallel to the floor, open palm)
9		Int	Pointing (with index finger) of something straight ahead (with outstretched arm)
10		Int	Silence gesture (bringing the index finger, with the remainder of the hand closed, on the lips)
11	2	Tr	Reach and grasp a small suitcase from the handle, lift it and place it on the floor (close to own chair, along own sagittal plane)
12	3	Tr	Reach and grasp a glass, drink for 3 seconds and place it in the initial position
13	4	Tr	Reach and grasp a phone receiver, carry it to own ear for 3 seconds and place it in the initial position
14	6	Tr	Reach and grasp a book (placed overhead on a shelf), put in on the table and open it (from right side to left side)
15	8	Tr	Reach and grasp a small cup from the handle (2 fingers + thumb), drink for 3 seconds and place it in the initial position
16	11	Tr	Reach and grasp an apple, mimic biting and put it in the initial position
17	12,13	Tr	Reach and grasp a hat from its top and place it on own head
18	12	Tr	Reach and grasp a cup from its top, lift it and put it on the left side of the table
19	15	Tr	Receive a tray (straight ahead, with open hand) and put it in the middle of the table
20	16	Tr	Reach and grasp a key in a lock (vertical axis), extract it from the lock and put it on the left side of the table
21	1	T-M	Reach and grasp a bottle, pour water into a glass and put the bottle in the initial position
22	2,3,4	T-M	Reach and grasp a tennis racket (placed along own frontal plane) and play a forehand (the subject is still seated)
23	5	T-M	Reach and grasp a toothbrush, brush teeth (horizontal axis, one time left-right) and put it inside a holder (on the right side of the table)
24	6	T-M	Reach and grasp a laptop, open it (without changing its position) (4 fingers + thumb)
25	7,8,9	T-M	Reach and grasp a pen (placed on the right side of the table) and draw a vertical line on the table (from the top to the bottom)
26	7	T-M	Reach and grasp a pencil (placed along own frontal plane) (3 fingers + thumb) and put it inside a squared pencil holder (placed on the left side of the table)
27	9	T-M	Reach and grasp a tea bag in a cup (1 finger + thumb), remove it from the cup and place it on the table on the right side of the table
28	10	T-M	Reach and grasp a doorknob, turn it clockwise and counterclockwise and open the door
29	13	T-M	Reach and grasp a tennis ball (with fingertips) and place it in a basket on the floor (right)
30	14	T-M	Reach and grasp a cap (2 fingers + thumb) of a bottle (held by left hand), unscrew it and place it overhead on a shelf

It is worth noticing that, considering our optimization routine with the Obstacle Avoidance (OA) part, the minimum distance between the dummy manipulator and the obstacle is always greater than the radius of the obstacles (whose dimensions and position with respect to the Inertial System of Reference – see Fig. 3 – are reported in Table 2). This does not apply when the OA part is not implemented (i.e. free motion case).

**Fig. 11:**
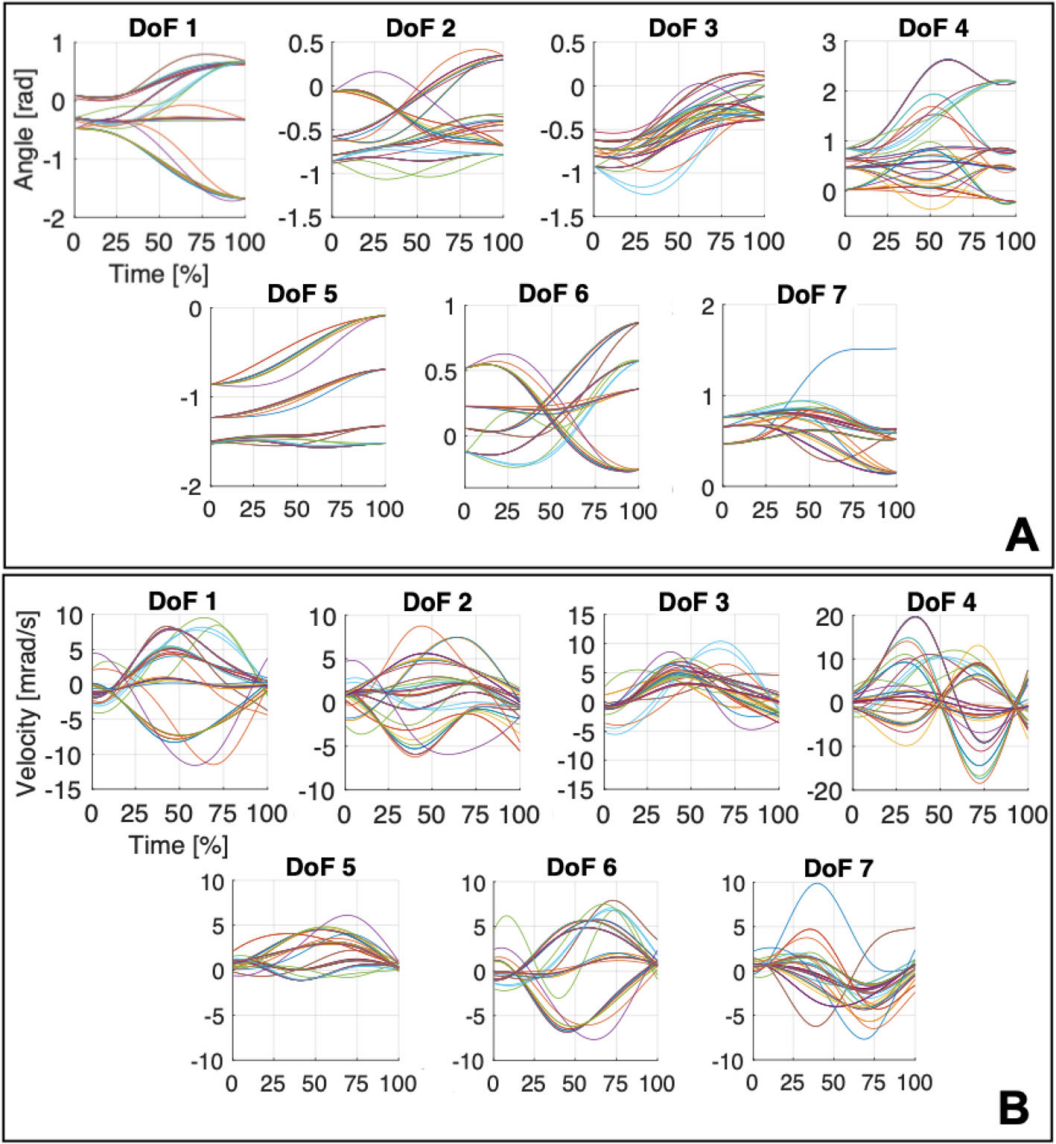
Angular positions and velocities for a set of simulations performed on movements T.1, T.2, T.3 and T.4 with different obstacles. In Subfigure A, we report the angular positions for the seven DoFs, arranged following the order of Fig. 4, while in Subfigure B we show the corresponding angular velocities

In the considered simulations, the first fPC alone accounts for the 96% of the cost reduction, while - with only the first three functional Principal Components - 100% reduction of the cost is achieved. This is coherent with the results in Table 3, and confirms the effectiveness of the proposed strategies for selecting the number of functional Principal Components to be used. Additional experiments have been performed to show how the algorithm works in case of obstacles close to the elbow trajectory. We reported in Fig. 11 results for tasks 1 and 2. The interested reader is invited to refer to the attached video from different points of view 1.

#### Human-Likeliness of trajectories

The method proposed in this work embeds human-like profiles for upper-limb movements without explicitly constraint this behavior in the optimization problem. As discussed previously, it has been observed that human movements are typically characterized by i) bell-shapes of velocity profiles [3, 4]; ii) minimization of jerk values [3]. For this reason, in this work we rely on this observation to verify the human-likeliness of the trajectories generated though our method.

Regarding the velocity profiles, in Fig. 10, we report the angular profiles and the corresponding velocities, for all the considered simulations. The bell-shaped distributions are effectively embedded in the considered evolutions, not only in the case of point-to-point free motion but also while avoiding contact with one or more obstacles. Moreover, the norm of the trajectories jerk is always lower than 5 10^−7^rad (7.92 10^−8^±8.53 10^−8^rad), in line with the observations of [5]. Finally, all the evolutions of angular velocity present a marked bell-shaped profile, as observed in [4]. Few exceptions can be evidenced mostly for the elbow joint (dof 4), which however can be seen as a sequence of two bell shaped trajectories, one for the opening and one for the closing.

## Discussions

In this paper, we presented a novel method to endow robotic manipulators with human-like movements by directly using human principal motion modes identified through functional analysis. In this way, human-likeness is intrinsically guaranteed.

This leaves room to optimize other aspects related to motion generation, such as obstacle avoidance - as done in this work - energetic consumption, joint torques, time efficiency and so on. It is worth noticing that the aim of our work is not to solve planning problems but instead to propose a new method for intrinsically embedding human-likeness in motion generation. Future work will encompass the evaluation with sophisticated planning problems and state of the art planning benchmarks.

In the general case, the resulting optimization problem deals with a non-linear cost function which may present local minima. The current implementation relies on the MATLAB optimization routine fmincon, which implements a gradient-based local optimization. This does not necessarily guarantee the convergence to the global optimum, for which the use of global optimization algorithms would be needed and will be considered as future work. Moreover, any comparison with other existing algorithms in terms of execution time is beyond the scope of this work. Nevertheless, we do believe that the proposed method could also enable a strong reduction of the computational costs w.r.t. state of the art algorithms, since the optimization space is constrained to a reduced set of parameters (i.e. the principal function weights) instead of the whole evolution of the robot pose. Future work will focus on more efficient implementation of the proposed algorithm.

Note that the method is specifically tailored on systems having the same kinematic structure of the human upper limb i.e the kinematic model used to describe human upper limb. The major limitation of this specific implementation is that the direct generalization to other kinematics is not straightforward and would require to solve a mapping problem. A solution could be to select a mapping policy as done in [46]. An approach based on Impedance Control [47] could also be used to define a framework for which the trajectories generated with our approach can be used as a reference for the controller of a generic anthropomorphic manipulator (see [48].)

## Conclusions

The methodology presented in this paper is designed to easily and uniquely generate human-like trajectories. We do believe that this work may have a major impact for the control of active devices during the interaction with humans. This is particularly relevant for the applications in which the robot is used as an instrument for human assistance or rehabilitation. Indeed, human-like robotic platforms that interact with humans, for example for self-feeding or elderly care, could increase their effectiveness, relying on the acceptability and predictability of the generated movements. The potential translational impact of our method in the field of companion robots could be also extremely significant. Indeed, the implementation of human-inspired point-to-point trajectories fully meets one of the three main envisioned requirements that companion robots are expected to fulfil in the future, which is related to the need for smooth robotic motion patterns (see [49] for further details).

Applications for rehabilitation devices are finally envisioned. Indeed, our strategy could be used to suggest preferred movements for the rehabilitation exoskeletons, in conjunction with an analogous modulation of the robot impedance. In our future work we will investigate the extension of this approach to the modulation of the impedance parameters. We believe that this could provide more therapeutic opportunities and enable novel approaches in rehabilitation.

## List of actions

Table 5 lists the 30 actions considered in the protocol of this study. For each row, the first element counts the task number, the second links to the grasp taxonomy propose by Cutkosky in [50], the third indicates the class of movement, and the fourth reports a brief description of the task. The considered actions are also pictured in Fig. 1. These movements are clustered in three classes, depending on the interaction with the external environment: intransitive, which collects gestures and actions that do not require the use of an object; transitive, in which an object is used to complete the task; tool-mediated, in which the actions require an object to interact with another object.

## Data Availability

Data used for this work will be published and made freely available as part of a larger multi-center dataset.

## Abbreviations

ADLs: Activities of daily living
fPCA: Functional principal component analysis
fPCs: Functional principal components
HL: Human-likeliness
HRI: Human-robot interaction
FK: Forward kinematics
OA: Obstacle avoidance

## Footnotes

1 https://youtu.be/Grg4L4fljdg
